# Association between Tfh and PGA in children with Henoch–Schönlein purpura

**DOI:** 10.1515/med-2021-0318

**Published:** 2021-07-02

**Authors:** Miao Meihua, Li Xiaozhong, Wang Qin, Zhu Yunfen, Cui Yanyan, Shao Xunjun

**Affiliations:** Department of Clinical Laboratory, Children’s Hospital of Soochow University, Suzhou 215025, China; Department of Immunology, School of Biology & Basic Medical Science, Soochow University, Suzhou 215021, China

**Keywords:** Henoch–Schönlein purpura, follicular helper CD4^+^ T cells, α-1,4-d-polygalacturonic acid, immunity

## Abstract

**Objective:**

The aim of this study was to investigate the roles of follicular helper CD4^+^ T cells (Tfh) and serum anti-α-1,4-d-polygalacturonic acid (PGA) antibody in the pathogenesis of Henoch–Schönlein purpura (HSP).

**Methods:**

ELISA was performed to determine serum PGA-IgA and PGA-IgG. Flow cytometry was utilized to determine the peripheral CD4^+^ CXCR5^+^ and CD4^+^ CXCR5^+^ ICOS^+^ Tfh cells. Real-time PCR was conducted to determine the expression of Bcl-6 gene. Then the change of Tfh cells was analyzed, together with the association with the anti-PGA antibody as well as the roles in the pathogenesis of HSP.

**Results:**

Compared with the cases with acute respiratory infection and elective surgery, the proportion of CD4^+^ CXCR5^+^ and CD4^+^ CXCR5^+^ ICOS^+^ Tfh cells in the HSP group showed significant elevation (*P* < 0.001). A significant correlation was noticed between PGA-IgA and CD4^+^ CXCR5^+^ Tfh cells (*r* = 0.380 and *P* = 0.042) and CD4^+^ CXCR5^+^ ICOS^+^ Tfh cells (*r* = 0.906 and *P* < 0.001). The expression of Bcl-6 in the HSP group showed no statistical difference compared with that in the acute respiratory infection and the surgery control (*P* < 0.05).

**Conclusion:**

Increased activity of Tfh cells, which is closely related to mucosal immunity, may be a major contributor in the elevation of PGA-IgA, and Tfh cells and PGA-IgA are closely related to the occurrence of HSP.

## 1 Introduction

Henoch–Schönlein purpura (HSP), now known as IgA vasculitis, is a common systemic disease in children. A large number of circulating immunocomplexes containing IgA antibodies are deposited on the wall of small vessels in the affected organs or tissue, and causing small vasculitis mediated by complement-induced immune inflammatory responses is the main feature of HSP [1,2,3]. However, its pathogenesis is still unclear, including the source of IgA antibodies, especially its specific target antigens [4].

IgA was presented *in vivo* in a form of monomer and polymer. In the mucous tissues, IgA was mainly in a polymer form, while in the peripheral circulation, monovalent forms account for more than 90% [5]. It is worth noting that the IgA-type antibodies associated with pathogenesis in the peripheral circulation of patients with nephritis-type HSP or IgA nephritis are mainly in the form of multimers [6,7], and the cells secreting the corresponding IgA-type antibodies are present in the bone marrow, tonsils and lymphatic follicles in the intestinal mucosa, along with the increase in number [8,9]. Tonsillectomy can significantly reduce serum IgA-type antibody levels [9]. These findings suggest that mucosal B cells migrate to systemic immune sites such as the bone marrow, followed by abnormal homing, which continue to produce their “correct” mucosal IgA at new locations, resulting in increased levels of poly-IgA in serum [10,11]. Therefore, mucosal infection and its immune response are considered to be potential mechanisms for elevated serum IgA in HSP [12].

Follicular helper CD4^+^ T cells (Tfh) are the major regulatory cell for the secretion of IgA in mucosal immunity, while the differentiation and function maintenance of Tfh cells are dependent on the transcription factor Bcl-6 [13,14]. Also, these cells are crucial for the regulation of the germinal center, which can promote the formation of plasmocytes and long-term memory B lymphocytes [14]. Tfh cells are identified by CD4^+^ CXCR5^+^ CD markers and are characterized by immunoregulatory molecules such as inducible T cell co-stimulator (ICOS), programmed cell death protein-1, etc. Circulating CD4^+^ CXCR5^+^ Tfh cells express low levels of Bcl-6 and ICOS, but ICOS can be further activated and enhanced; therefore, circulating CD4^+^ CXCR5^+^ ICOS^+^ Tfh cells reflect activated Tfh cells [15,16]. However, the roles of Tfh in the modulation of B lymphocytes in the HSP cases are still unclear except for reports from rare studies [17]. Therefore, it is necessary to investigate the roles of Tfh cells in the pathogenesis of HSP, especially the relationship with the specific increase in IgA antibodies.

Recently, we reported the presence of specific antibodies against α-1,4-d-polygalacturonic acid (PGA), a main component of pectin, in non-nephritis-type HSP patients, especially PGA-IgA [18]. This study was to further analyze the changes of Tfh cells in children with acute HSP without renal impairment, and the correlation between Tfh and anti-PGA antibodies.

## 2 Patients and methods

### 2.1 Study population

According to the classification criteria of pediatric vasculitis developed by the European Union against rheumatology and the European Society of Pediatrics Rheumatology in 2006 [19], 29 cases of HSP in acute stage (male: 15, female: 14, mean age: 5.06 ± 3.25 years and range: 1–13 years), manifested as typical skin rash, abdominal pain (such as gastrointestinal symptoms and vomiting), arthralgia, etc., were collected from outpatient and inpatient children without renal damage (no microscopic hematuria, immunoglobulin G, α1 microglobulin, β2 microglobulin, urinary transferrin, *N* acetyl glucosaminidase and normal urine microalbumin). Meanwhile, matched control consisted of 28 children (male: 15, female: 13, mean age: 5.34 ± 6.94 years and range: 1–12 years) with acute respiratory infection, 30 cases received elective surgery including hernia, circumcision or polydactyly (male: 16, female: 14, mean age: 4.64 ± 4.02 years and range: 1–14 years).


**Ethics approval and consent to participate:** The study protocols were approved by the Ethical Committee of Children’s Hospital of Soochow University.
**Consent to publish:** Written informed consent for the publication has been obtained from the patients. Consent for publication was also obtained from the patient’s family.

### 2.2 Sample collection

Peripheral venous blood samples (3–5 mL) were collected from each participant. Samples were placed at room temperature and Tfh cells were detected within 4 h of sample collection. Serum was also separated within 4 h of sample collection and stored at −80°C prior to analysis, and repeated freeze-thaw cycles were avoided.

### 2.3 Serum PGA-IgA and PGA-IgG determination

ELISA was performed to determine serum PGA-IgA and PGA-IgG according to the method established previously [18].

### 2.4 Flow cytometry

Flow cytometry was used to determine the surface marker expression, including CD4^+^ CXCR5^+^ and CD4^+^ CXCR5^+^ ICOS^+^ Tfh lymphocyte subsets. Add 100 μL of whole blood (lithium heparin) and 10 μL each of different fluorescein-labeled monoclonal antibodies (BD), including CD4-FITC, CXCR5-APC and ICOS-PerCP-CY5, to one flow-type special test tube sequentially, mix well and store in the dark at room temperature for 15 min. Follow-up operations, including addition of 500 µL of hemolytic agent (BD), were carried out according to the manufacturer’s instructions and detected on the flow cytometer (FACS Calibur, BD). CD4-SSC was used for gating CD4^+^ cells, then CD4-CXCR5 was used for gating CD4^+^ CXCR5^+^ Tfh from CD4^+^ cells and CXCR5-ICOS was used for gating CD4^+^ CXCR5^+^ ICOS^+^ Tfh cells from CD4^+^ CXCR5^+^ cells. BD Multiset Software was used for the subset analysis. Before the patient samples were detected, the calibration of the microspheres (CaliBRITE Beads) was used to control the optical quality and fluorescence compensation of the flow cytometry to ensure that the indicators of the instrument were within the allowable range of quality control.

### 2.5 Real-time PCR

Total RNA was extracted from mononuclear cells using commercial Kit (QIAGEN), and mononuclear cells were obtained by density gradient centrifugation (Fluid Ficoll-Paque, GE). The specific operations were carried out according to the manufacturer’s instructions. The cDNA synthesis was carried out with approximately 2 µg RNA using MMLV enzyme (Invitrogen). Real-time PCR was conducted using SYBR Green (ROCHE) on a LightCycler 480II system with the primers for Bcl-6 (forward primer: 5′-CATGCAGAGATGTGCCTCCACA-3′; reverse primer: 5′-TCAGAGAAGCGGCAGTCACACT-3′). The mRNA level was normalized by beta-actin. PCR reactions were performed in a total volume of 10 µL containing 5 µL of 2× SYBRPremix (Roche), 0.2 µL of each specific primer to a final concentration of 200 nM, and 1 µL of cDNA template. The PCR conditions consisted of denaturation at 95°C for 5 min, followed by 40 cycles of denaturation at 95°C for 10 s, annealing at 58°C for 20 s and extension at 72°C for 30 s. Using the average ∆Ct (Ct_Bcl-6_ – Ct_actin_) in 30 surgical control samples as a reference, the relative expression of Bcl-6 in each sample was calculated as 2^−ΔΔCt^.

### 2.6 Statistical analyses

Data analyses were performed using SPSS software, version 22.0 (IBM, Armonk, NY, USA). Between-group comparisons were performed using independent samples Kruskal–Wallis one-way analysis of variance. Spearman’s rank correlation coefficient was used to analyze the correlation between the measurement data that were not normally distributed. A *P* value < 0.05 was considered to be statistically significant.

## 3 Results

### 3.1 The proportion of Tfh cells showed a significant increase in HSP cases

**Figure 1:**
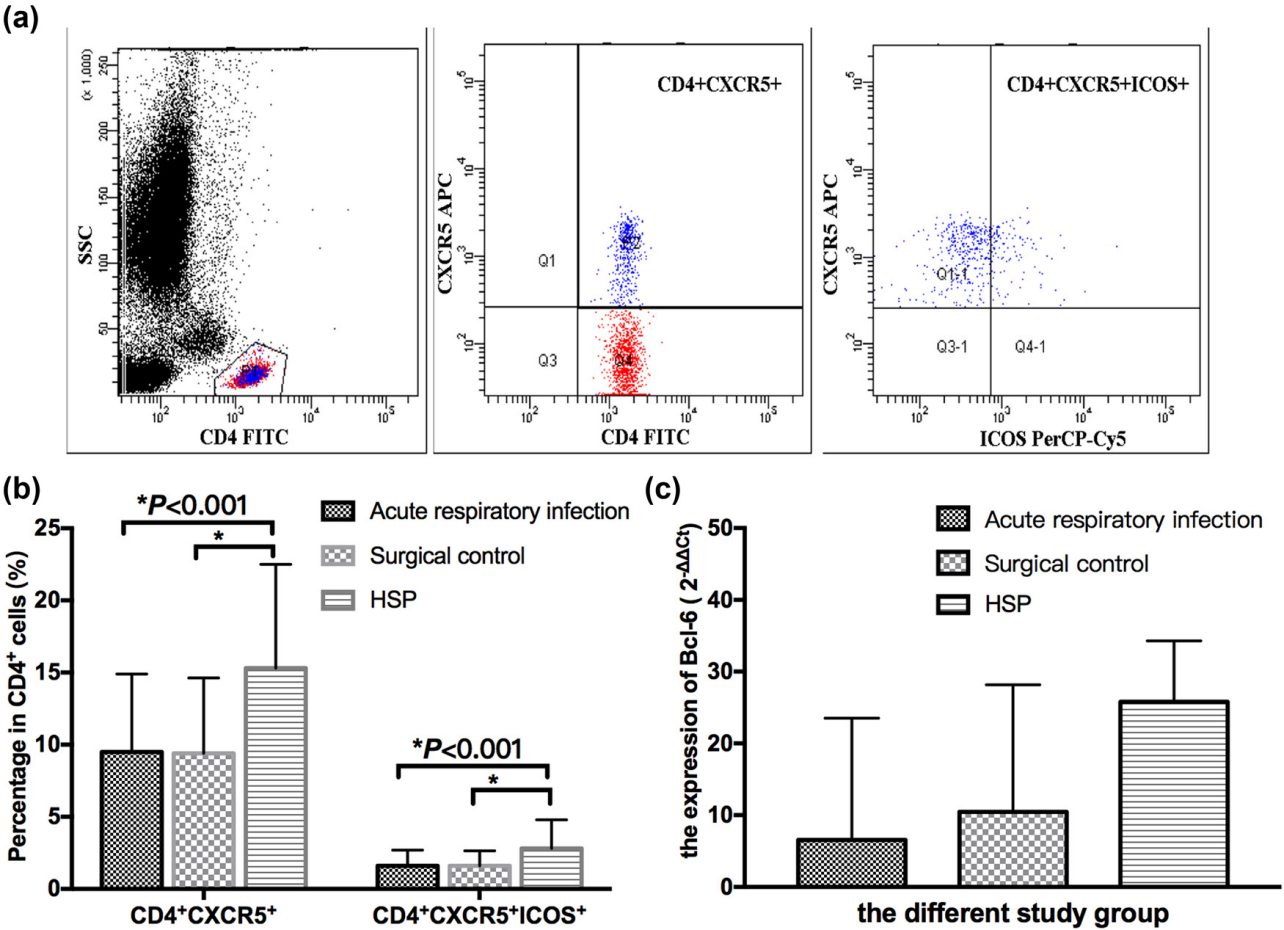
CD4^+^ CXCR5^+^, CD4^+^ CXCR5^+^ ICOS^+^ Tfh cells and Bcl-6 expression in the HSP children and control group. (a) The gating strategy for Tfh cell analysis, and the percentages of CD4^+^ cells, CD4^+^ CXCR5^+^ and CD4^+^ CXCR5^+^ ICOS^+^ Tfh cells were obtained in sequence. (b) The proportion of CD4^+^ CXCR5^+^ and CD4^+^ CXCR5^+^ ICOS^+^ Tfh cells was significantly higher than that of the acute respiratory tract infection and surgical control group (*P* < 0.001). No statistical differences were noticed in the proportion of CD4^+^ CXCR5^+^ and CD4^+^ CXCR5^+^ ICOS^+^ Tfh cells between the acute respiratory tract infection and surgical control (*P* > 0.05). (c) No statistical differences were noticed in the Bcl-6 expression among the acute respiratory infection group, surgical control and HSP groups (*P* > 0.05).

The gating strategy for Tfh cell analysis is shown in Figure 1a, and the percentages of CD4^+^ cells, CD4^+^ CXCR5^+^ and CD4^+^ CXCR5^+^ ICOS^+^ Tfh cells were obtained in sequence. In children with HSP, the proportion of CD4^+^ CXCR5^+^ and CD4^+^ CXCR5^+^ ICOS^+^ Tfh cells were significantly higher than that of the acute respiratory tract infection and surgical control group (*P* < 0.001). No statistical differences were noticed in the proportion of CD4^+^ CXCR5^+^ and CD4^+^ CXCR5^+^ ICOS^+^ Tfh cells between the acute respiratory tract infection and surgical control group (*P* > 0.05, Figure 1b). The expression of Bcl-6 in the HSP group showed a strong tendency of higher expression, but no statistical differences compared with the acute respiratory tract infection and surgical control group (*P* > 0.05, Figure 1c).

### 3.2 The proportion of Tfh cells was significantly correlated with PGA-IgA in HSP cases

**Figure 2:**
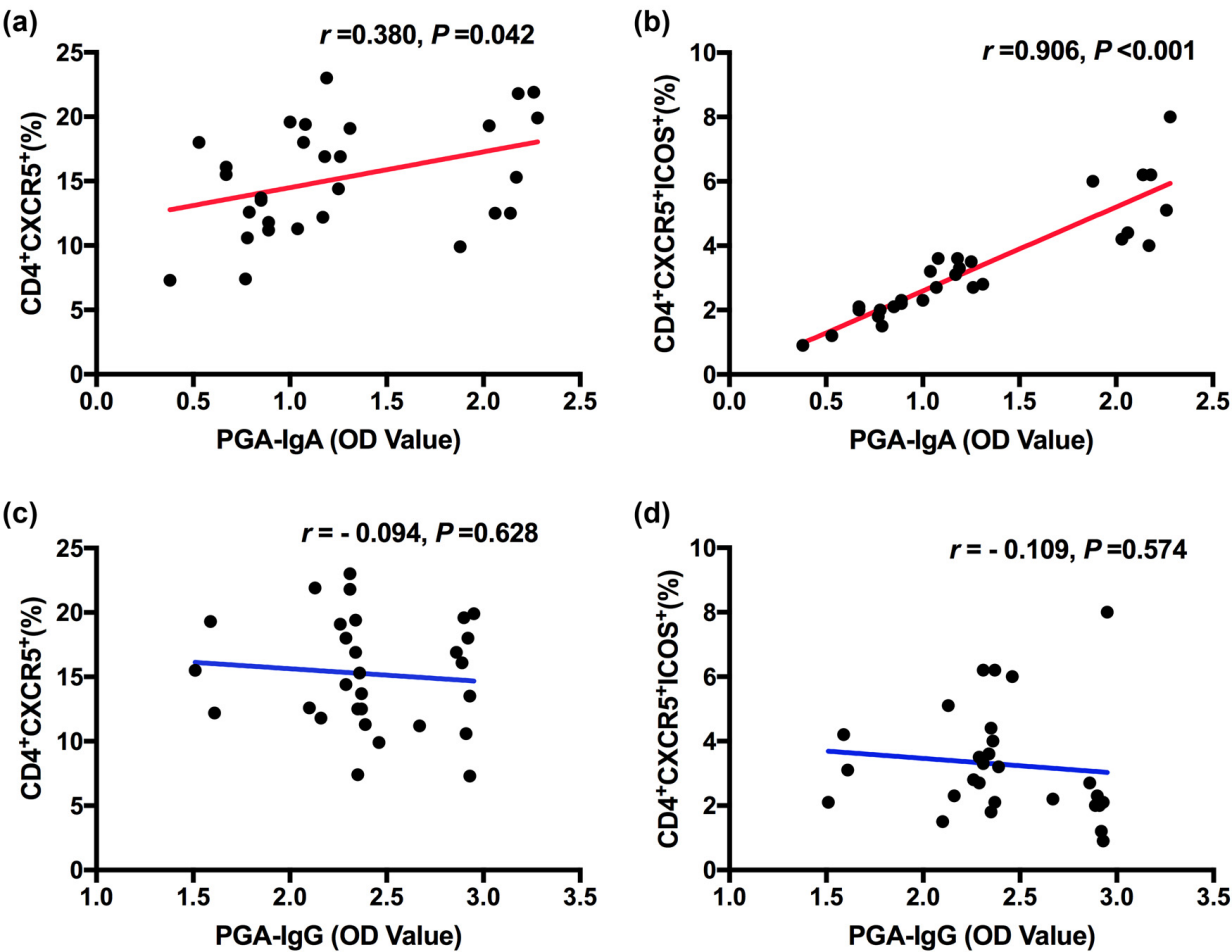
Correlation between PGA-IgA, PGA-IgG and CD4^+^ CXCR5^+^, CD4^+^ CXCR5^+^ ICOS^+^ Tfh in HSP children. (a and b) Correlation analysis between PGA-IgA and CD4^+^ CXCR5^+^, CD4^+^ CXCR5^+^ ICOS^+^ Tfh. (c and d) Correlation analysis between PGA-IgG and CD4^+^ CXCR5^+^, CD4^+^ CXCR5^+^ ICOS^+^ Tfh.

For the association between Tfh cell proportion and anti-PGA antibody, PGA-IgA was correlated with the CD4^+^ CXCR5^+^ (*r* = 0.380 and *P* = 0.042) and CD4^+^ CXCR5^+^ ICOS^+^ Tfh in the HSP group (*r* = 0.906 and *P* < 0.01, Figure 2a and b). No correlation was noticed between PGA-IgG and CD4^+^ CXCR5^+^ and CD4^+^ CXCR5^+^ ICOS^+^ Tfh in the HSP group (Figure 2c and d). No correlation was noticed between PGA-IgG/PGA-IgA and CD4^+^ CXCR5 ^+^/CD4^+^ CXCR5^+^ ICOS^+^ Tfh in the acute respiratory tract infection group and the surgical control group.

## 4 Discussion

The main feature of HSP is that immune complexes containing IgA-type antibodies are deposited on the affected small vessel wall, and IgA may be in the monomeric or multimeric form, while their multimeric forms may be associated with mucosal immune abnormalities [5,6,7,9]. In this study, CD4^+^ CXCR5^+^ and CD4^+^ CXCR5^+^ ICOS^+^ Tfh cells were abnormally elevated in children with non-nephritis-type HSP, and were significantly associated with PGA-IgA, especially CD4^+^ CXCR5^+^ ICOS^+^-activated Tfh cells.

In the mucosal immunity, Tfh cells are the major component in the activation of plasmocytes secreting IgA. Tfh cells can promote B cell activation and differentiate into plasma cells by secreting IL-21 and interacting directly with B cells, while excessive activation of Tfh cells breaks the balance among immunoregulatory cells, such as Tfh cells and Treg cells, thereby enhancing plasma cell activation, which leads to a large secretion of IgA [20]. The predominance of Th2 cells, the imbalance of Th2, Th17 and Treg cells interacting with IgA-secreting plasmocytes, etc., has been well established in the pathogenesis of HSP [21,22,23,24]. In-line with the previous study [17], our data showed specific elevation of CD4^+^ CXCR5^+^ and CD4^+^ CXCR5^+^ ICOS^+^ Tfh cells in HSP cases. Besides, the differentiation of Tfh requires the Bcl-6 that can inhibit the differentiation of the other T lymphocyte subsets, including Th1, Th2 and Treg [13]. Meanwhile, Bcl-6 could contribute to the expression of CXCR5, which resulted in homing of lymphocytes into the B-cell follicles [25]. Therefore, we also tested the expression differences of Bcl-6 in different study groups. The results showed that compared with children with elective surgery and respiratory infections, the HSP group had a strong tendency of higher expression of Bcl-6, while no statistical significances were shown. On one hand, non-parametric statistical methods may reduce the power of the test. On the other hand, we only obtained mononuclear cells, but no T cells or Tfh cells. However, the strong increasing trend of Bcl-6 gene expression in the HSP group, which was consistent with the increase in Tfh and activated Tfh, suggests their role for the pathogenesis of HSP.

In addition, our results found that PGA-IgA was significantly associated with CD4^+^ CXCR5^+^ Tfh cells, particularly CD4^+^ CXCR5^+^ ICOS^+^-activated Tfh cells, strongly suggesting that the increase in the proportion of CD4^+^ CXCR5^+^ ICOS^+^-activated Tfh cells may be responsible for the elevation of PGA-IgA antibody. PGA is the main component of pectin, and pectin is commonly used in the food, pharmaceutical and cosmetic industries. Patients with HSP may have high reactivity of T and B lymphocytes in the intestinal mucosa to PGA-specific antigen stimulation, enhancing the specific proliferation of IgA-producing B cells, and abnormal homing, leading to elevation of Poly-IgA production [6,26]. Meanwhile, as the major cause for HSP, upper respiratory tract infection together with aberrant activation of mucosal immunity in the tonsils, especially microbial infection with cross-reactive antigens in PGA, may trigger proliferation and aberrant homing of the B lymphocytes secreting IgA in tonsils, which then promoted the pathogenesis of HSP. Interestingly, the PGA-IgA level in the cases with acute respiratory tract infection was significantly higher than that of the surgical control [18], while no statistical differences were noticed in the Tfh cells between these two groups. This demonstrated that besides the sample factor, the inflammatory environments of the Tfh cells may affect the generation of PGA-IgA antibodies.

Indeed, the immune system is a complex system, and the mechanism of PGA-IgA and Tfh cell’s elevation remains to be further studied, including confirmation of whether PGA-IgA is monomeric or multimeric in plasma as well as the presence of specific proliferation and homing abnormalities of mucosal plasmocytes secreting IgA, especially with animal models. The current findings support an increase in PGA-IgA, and increased Tfh cell activation is an important cause of HSP in children, but its role remains to be further accumulated.
